# Texture Analysis in the Diagnosis of Primary Breast Cancer: Comparison of High-Resolution Dedicated Breast Positron Emission Tomography (dbPET) and Whole-Body PET/CT

**DOI:** 10.3389/fmed.2020.603303

**Published:** 2020-12-23

**Authors:** Yoko Satoh, Kenji Hirata, Daiki Tamada, Satoshi Funayama, Hiroshi Onishi

**Affiliations:** ^1^Yamanashi PET Imaging Clinic, Yamanashi, Japan; ^2^Department of Radiology, University of Yamanashi, Yamanashi, Japan; ^3^Department of Diagnostic Imaging, School of Medicine, Hokkaido University, Sapporo, Japan

**Keywords:** dedicated breast positron emission tomography (dbPET), positron emission tomography/computed tomography (PET/CT), texture analysis, breast cancer, 18F-FDG

## Abstract

**Objective:** This retrospective study aimed to compare the ability to classify tumor characteristics of breast cancer (BC) of positron emission tomography (PET)-derived texture features between dedicated breast PET (dbPET) and whole-body PET/computed tomography (CT).

**Methods:** Forty-four BCs scanned by both high-resolution ring-shaped dbPET and whole-body PET/CT were analyzed. The primary BC was extracted with a standardized uptake value (SUV) threshold segmentation method. On both dbPET and PET/CT images, 38 texture features were computed; their ability to classify tumor characteristics such as tumor (T)-category, lymph node (N)-category, molecular subtype, and Ki67 levels was compared. The texture features were evaluated using univariate and multivariate analyses following principal component analysis (PCA). AUC values were used to evaluate the diagnostic power of the computed texture features to classify BC characteristics.

**Results:** Some texture features of dbPET and PET/CT were different between Tis-1 and T2-4 and between Luminal A and other groups, respectively. No association with texture features was found in the N-category or Ki67 level. In contrast, receiver-operating characteristic analysis using texture features' principal components showed that the AUC for classification of any BC characteristics were equally good for both dbPET and whole-body PET/CT.

**Conclusions:** PET-based texture analysis of dbPET and whole-body PET/CT may have equally good classification power for BC.

## Introduction

Due to the recent advances in BC treatment, neo-adjuvant systemic chemotherapy is often performed before surgery. Therefore, highly accurate staging before treatment is essential. However, because BC is characterized by heterogeneity, it is difficult to predict tumor characteristics and prognosis from small specimens biopsied from a limited number of lesions. 18F-fluorodeoxyglucose (FDG) positron emission tomography (PET) can assess metabolic information on various tumors—a difficult task with conventional imaging modalities—and evaluate a wide range of pathological conditions in a minimally invasive manner. For this reason, PET is now widely used for benign vs. malignant lesion differentiation, staging, recurrence diagnosis, and prediction of prognosis in various types of cancer, including BC (1).

Until recently, the usefulness of local evaluation by whole-body PET/computed tomography (CT) has been limited by its limited spatial resolution and the physiological background accumulation in mammary glands (2). However, in more recent years, the performance of whole-body PET/CT scanners has increased along with their diagnostic ability in local evaluation due to the widespread use of devices using time-of-flight (TOF) or point-spread-function (PSF) methods for image reconstruction (3). Furthermore, high-resolution breast PET scanners have been developed to detect BC lesions smaller than those detectable by whole-body PET (4–6). By using the two devices in combination, it has been possible to accurately and more rapidly evaluate local and metastatic BC lesions.

In the past few years, research on radiomics has focused particularly on texture analysis using various imaging modalities, including BC studies utilizing MRI and US (7, 8). Some studies reported that PET images' texture features are associated with BC subtypes and prognosis (9–11). Texture analysis, which assesses intra-tumoral heterogeneity to compute image-specific information, is highly reproducible, has little variation among diagnostic radiologists, and will help mitigate the shortage of said radiologists who are excellent at diagnosing BC imaging. Its usefulness suggests that PET may contribute more widely to the diagnosis, treatment, and post-treatment management of BC.

Regarding the comparison between dbPET and whole-body PET/CT, there have been reports on the evaluation of the standard performance of the scanners and detectability of BC (12, 13), but none on the comparison of their diagnostic ability using texture analysis. This study aimed to compare the classifying ability of PET-derived texture features for BC's tumor characteristics between dbPET and whole-body PET/CT.

## Materials and Methods

This single-institute, retrospective study was approved by the Institutional Review Board and the Ethics Committee of our institute and was carried out in accordance with the Declaration of Helsinki. Written informed consent for future access and anonymous use of their data was obtained from each patient.

### Patients

We enrolled 798 consecutive women who underwent dbPET and whole-body PET/CT at our institute between April 2015 and March 2018. BCs that were selected fulfilled the following inclusion criteria: (1) the molecular subtype of BC had been determined; (2) patient clinical history was available. The exclusion criteria were as follows: (1) patients with a history of other malignancies; (2) missing or incomplete clinical data; (3) patients undergoing chemotherapy or within 1 year after its completion; (4) BC not successfully extracted in both dbPET and PET/CT because the SUV of the background mammary gland was higher than 40% of the BC SUVmax.

### Subtype Classification

BC diagnoses through tumor histology and immunohistochemistry were made using surgical or biopsy specimens of core needle biopsy before neoadjuvant chemotherapy. Tumor, lymph nodes, and metastasis (TMN) categorization of malignant tumors was established following the 8th edition of the American Joint Committee on Cancer (AJCC) staging system (14). The molecular markers examined included estrogen receptor (ER), progesterone receptor (PR), and human epidermal growth factor 2 amplified (HER2) expression. ER and PR status was considered positive for tumors showing at least 10% of positive cells. HER2 status was assessed by immunohistochemistry (IHC): Tumors scored as 3+ were classified as HER2 positive; those scored as 0 or 1+ were classified as HER2 negative. For tumors scored as 2+, further confirmation using molecular tests [*in situ* hybridization (ISH)] was obtained. ISH non-amplified tumors were classified as HER2 negative, and ISH amplified ones as HER2 positive. IHC classification followed the 13th St. Gallen International Breast Cancer Conference (2013) recommendations, with a Ki67 threshold of 20% (15). BCs were classified into four subtypes: (1) luminal A: ER and/or PR positive, HER2 negative, and low expression of Ki67 (<20%); (2) luminal B: (a) ER and/or PR positive, HER2 negative and high expression of Ki67 (20% ≤ ), or (b) ER and/or PR positive, HER2 positive; (3) HER2: ER and PR negative, and HER2 positive; and (4) triple-negative: ER, PR, and HER2 negative.

### Ring-Shaped dbPET Scanner

The ring-shaped dbPET scanner (Elmammo, Shimadzu Corporation, Kyoto, Japan) comprises a total of 36 detector modules (12 per ring) arranged in three continuous rings, has a diameter of 195 mm and axial length of 156.5 mm, and has depth-of-interaction measurement capability (16). The transaxial effective field-of-view (FOV) is 185 × 156.5 mm^2^. Each detector block consists of a four-layered 32 × 32 array of lutetium oxyorthosilicate crystals coupled to a 64-channel positron-sensitive photomultiplier tube via a light guide. Attenuation correction was calculated using a uniform attenuation map with object boundaries obtained from emission data (17). Scatter correction was performed using the convolution-subtraction method (18) with kernels obtained by background tail fitting. This scanner's characteristics and standard performance have been previously reported in detail (5).

### Whole-Body PET/CT Scanner

Whole-body PET/CT scans were obtained using a Biograph Horizon TrueV FDG-PET/CT system (Siemens Medical Solutions, Knoxville, TN, USA). This system has 52 detector rings consisting of 160 blocks. Each block containing an array of 13 × 13 lutetium oxyorthosilicate crystals (4 mm × 4 mm × 20 mm) covering an axial FOV of 221 mm and transaxial FOV of 690 mm. A CT scan was performed for attenuation correction (130 kV; 15–70 mA; tube rotation time, 0.6 s per rotation; pitch, 1; transaxial FOV, 700 mm; and section thickness, 5 mm).

### Data Acquisition and Image Reconstruction

All patients fasted for at least 6 h before administration of 18F-FDG (3 MBq/kg). Sixty min after the injection, patients underwent whole-body PET/CT scanning prior to dbPET. The PET/CT images were reconstructed using the ordered subset expectation maximization method and time-of-flight algorithm with 4 iterations and 10 subsets. The CT data were resized from a 512 × 512 matrix to a 180 × 180 matrix to match the PET data and construct CT-based transmission maps for attenuation correction of the PET data with a post-reconstruction smoothing Gaussian filter (5 mm FWHM). The voxel size was 4.11 x 4.11 x 5 mm^3^.

Approximately 90 min after FDG injection, after the whole-body PET/CT scan, dbPET scanning was performed for 7 min for each breast. The dbPET images were reconstructed using a three-dimensional list mode dynamic row-action maximum-likelihood algorithm with one iteration and 128 subsets, a relaxation control parameter of β = 20, and a matrix size in the axial view of 236 × 200 × 236. Reconstruction was done with a post-reconstruction smoothing Gaussian filter (1.17-mm FWHM). Attenuation correction using a uniform attenuation map with object boundaries obtained from the emission data was performed on phantom or clinical dbPET images, respectively. The convolution subtraction method was the scatter correction method used, with kernels obtained by background tailfitting (18). The voxel size was 0.78 × 0.78 × 2.34 mm^3^.

### Image Analysis

The SUV of each tumor was measured by a spherical volume of interest (VOI). The SUV was a dose- and body-weight-corrected value of tissue tracer concentration. The delineation method used a relative threshold set to 40% of the maximum standardized uptake value (SUVmax) in the lesion to identify the VOI according to a previous report (19). Compared with previous studies, metabolic tumor volume (MTV) and total lesion glycolysis (TLG) were calculated for reference. The MTV was defined as the VOI volume, and TLG was calculated by multiplying the MTV by the mean SUV (SUVmean). The SUVmean was defined as the average of all voxels in the VOI. All processes were performed using Metavol (PMID: 25162396).

### Texture Analysis

The SUV was resampled using 64 discrete values from the lowest to highest SUV. We used the PTexture package that we developed in a previous study (20). PTexture is a package using Python to compute texture features from voxel lists. The entire source codes of PTexture are available at https://github.com/metavol/ptexture. Further details regarding texture analysis have been previously reported (21). Texture features were computed only from PET images and not CT because dbPET is not attached to a CT.

### Statistical Analysis

Wilcoxon signed-rank test was used for the inter-group comparison of texture features for each BC characteristic. Because of the large number of texture features extracted from PET components and their high correlation with each other, feature reduction was performed by principal component analysis (PCA). PCA was performed on 38 texture features. Because PCA can extract features without reducing the number of features in advance, all the obtained texture features were used for PCA. The predictive performance of each feature in classifying patients according to Tumor (T)-category, Lymph node (N)-category, molecular subtype, and Ki67 level was evaluated and quantified using the area under the curve (AUC) in receiver-operating characteristic (ROC) analysis. A *p*-value <0.05 was considered to indicate statistical significance. JMP®15 (SAS Institute Inc., Cary, NC, USA) was used for the analyses.

## Results

### Patient Characteristics

**Figure 1:**
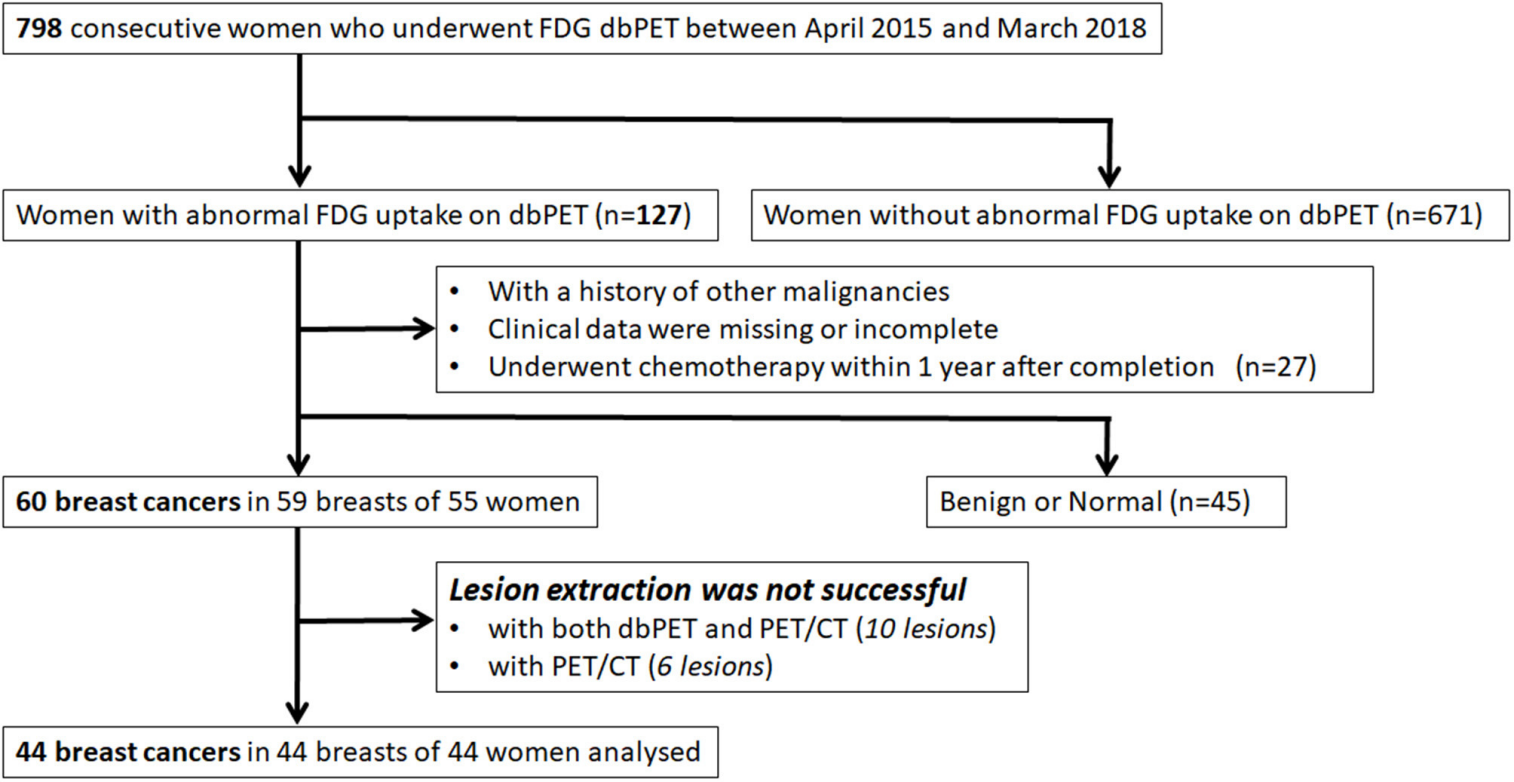
Inclusion and exclusion criteria. FDG, fluorodeoxyglucose; PET, positron emission tomography; dbPET, dedicated breast positron emission tomography; CT, computed tomography.

**Figure 2:**
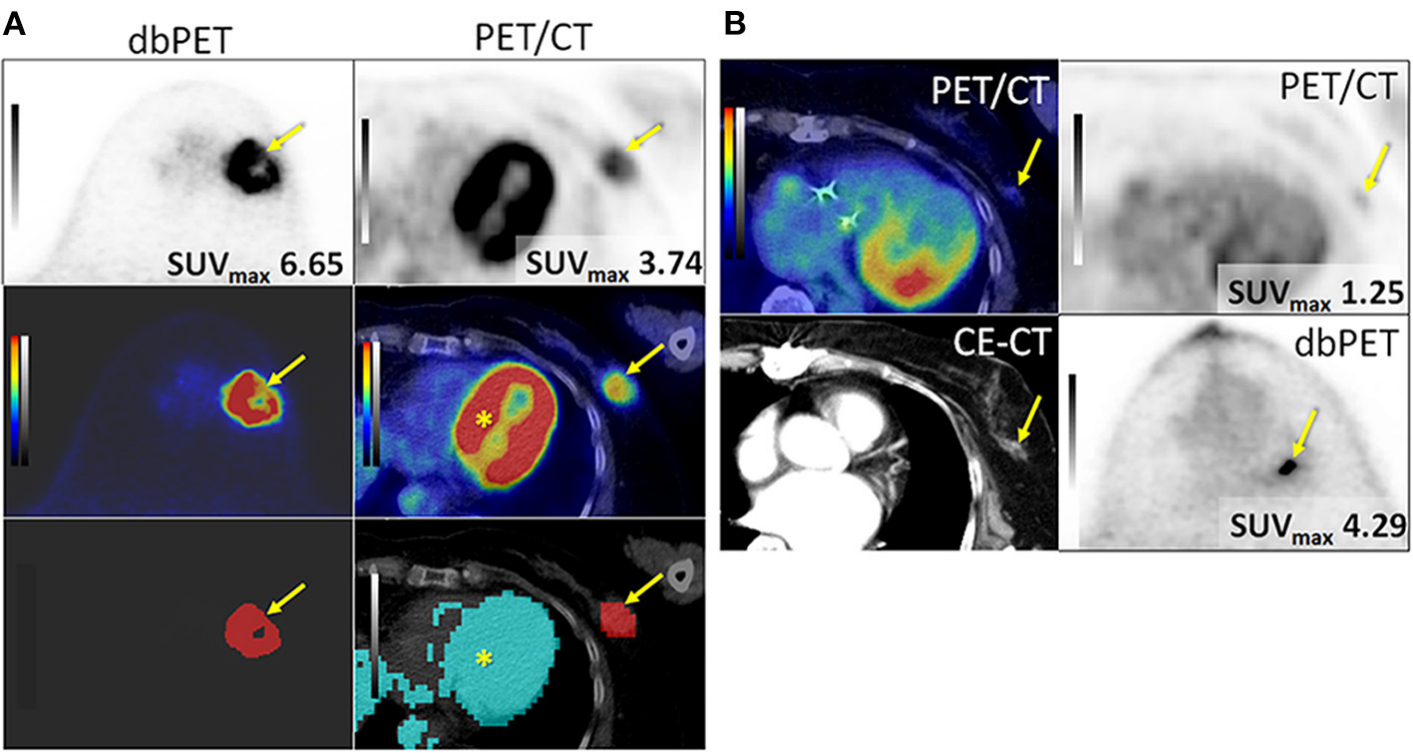
Successfully **(A)** and unsuccessfully **(B)** extracted breast cancers with dbPET and whole-body PET/CT. **(A)** Invasive ductal carcinoma in the left breast of an 84-year-old woman. BC (arrow) was successfully extracted with both dbPET and whole-body PET/CT (two images at the bottom) and clearly separated from the physiological uptake of the myocardium (*) with whole-body PET/CT. **(B)** Invasive ductal carcinoma in the left breast of a 74-year-old woman. It was successfully extracted with dbPET but failed with whole-body PET/CT; therefore, it was excluded from this study. BC, breast cancer; CE-CT, contrast-enhanced computed tomography; dbPET, dedicated breast positron emission tomography.

Of 798 enrolled consecutive women, 127 had abnormal findings on dbPET, and 60 BCs in 59 breasts of 55 women were histopathologically proven before or within 3 months after PET examination. Of 60 BCs that could be visually detected as showing abnormal FDG uptake with dbPET, 10 could not be identified with whole-body PET/CT. The visual detection rate of BC with PET/CT was 83% (50/60) of dbPET. Of 50 BCs that could be detected by both dbPET and whole-body PET/CT, 6 [ductal carcinoma *in situ* (DCIS): 1; T1b: 4; and T1c: 1] could not be successfully extracted with PET/CT. In addition, 10 (DCIS: 6; T1a: 2; T1b: 1 and T1c: 1) could not be successfully extracted with either dbPET or PET/CT because the lesion-to-background SUV ratio was low. Therefore, they were excluded from this study. Some BCs were false-negative with dbPET even in the FOV, but they could not be detected with whole-body PET/CT either. Finally, 44 BCs in 44 breasts of 44 women with a median age of 59 years (range: 37–87) were included in this study (Figure 1). Figure 2 shows representative BCs that were successfully extracted and failed to be extracted. Forty of these were invasive BCs, and four were non-invasive. Four, 9, 22, 8, and 1 BC patients were diagnosed with BC stage 0, I, II, III, and IV, respectively. Patient and tumor characteristics are shown in Table 1.

**Table 1:** Patient and tumor characteristics.

	**Number of lesions (*n* = 44) in 44 breasts of 44 patients**
Age, years (median, range)	59, 37–87
**Tumor (T)-category**	
Tis	4
T1	12
T2	25
T3	3
T4	0
**Lymph node (N)-category**	
N0	22
N1	13
N2	6
N3	3
**Stage**	
0	4
I	9
II	22
III	8
IV	1
**Histology**	
Non-invasive ductal	4
**Invasive carcinoma**	
Ductal	37
Lobular	1
Ductal and lobular	1
Apocrine	1
**Ki67 level**	
20%>	14
≥ 20%	26
Not specified	4
**Tumor subtype**	
Luminal A	19
Luminal B/HER2-	15
Luminal B/HER2+	2
HER2	3
Triple-negative	5

*HER2, human epidermal growth factor receptor 2 amplified*.

### Comparison of the Ability to Predict Tumor Characteristics Using Texture Features of dbPET and Whole-Body PET/CT

We calculated a total of 38 texture features. Five features were computed from a histogram. Four matrices, comprised of gray-level co-occurrence matrix (GLCM), gray-level run length matrix (GLRLM), gray-level zone size matrix (GLZSM), and neighborhood gray-level difference matrix (NGLDM), were generated. Thirty-one features were computed from these four matrices. The number of voxels and sum of SUV were added to the 36 calculated texture features, and a total of 38 features were finally used in the analysis. These texture features are generally applied to the previous PET studies of various cancers (21). Twenty-three dbPET and 17 PET/CT texture features were significantly different between the Tis-1 and T2-4 groups. In addition, four dbPET and four PET/CT texture features were significantly different between Luminal A and the other groups. Other texture features of dbPET or PET/CT were not associated with any BC characteristics (Table 2).

**Table 2:** Associations (*p-value*) between texture PET parameters and tumor characteristics.

	**dbPET**		**PET/CT**	
	**Tis-1 vs. T2-4**	**Luminal A vs. Others**	**Tis-1 vs. T2-4**	**Luminal A vs. Others**
Num of Voxels	NS	NS	0.0179	NS
SUV_sum_	NS	NS	0.0047	0.0284
SDhist	<0.0001	NS	NS	NS
Skewness	0.0003	NS	0.0084	NS
Kurtosis	<0.0001	NS	0.0104	0.0152
Energy_Hist_	0.0003	NS	<0.0001	NS
Entropy_Hist_	0.0006	NS	<0.0001	NS
Homogeneity_GLCM_	NS	NS	NS	NS
Energy_GLCM_	NS	NS	0.0157	NS
Correlation_GLCM_	NS	NS	0.0359	NS
Contrast_GLCM_	NS	NS	NS	NS
Entropy_GLCM_	<0.0001	0.0404	0.0157	NS
Dissimilarity_GLCM_	<0.0001	NS	NS	NS
SRE	0.0005	NS	NS	NS
LRE	<0.0001	NS	NS	NS
LGRE	<0.0001	NS	0.009	NS
HGRE	<0.0001	NS	NS	NS
SRLGE	<0.0001	0.0302	0.0073	NS
SRHGE	<0.0001	0.036	NS	NS
LRLGE	NS	NS	0.0097	NS
LRHGE	NS	NS	NS	NS
GLNUr	NS	NS	NS	NS
RLNU	NS	NS	0.0147	NS
RP	NS	NS	NS	NS
SZE	NS	NS	NS	NS
LZE	<0.0001	NS	NS	NS
LGZE	<0.0001	NS	0.0058	NS
HGZE	<0.0001	NS	NS	0.0302
SZLGE	<0.0001	NS	0.0128	NS
SZHGE	<0.0001	0.032	NS	NS
LZLGE	NS	NS	NS	NS
LZHGE	NS	NS	NS	0.0196
GLNUz	NS	NS	NS	NS
ZSNU	0.0073	NS	0.0084	NS
ZP	0.0318	NS	NS	NS
Coarseness_NGLDM_	0.0032	NS	NS	NS
Contrast_NGLDM_	<0.0001	NS	NS	NS
Busyness_NGLDM_	<0.0001	NS	0.0233	NS

*Numbers are p-values when there was a significant difference. NS not significant*.

*SUV, standardized uptake value; SUV_sum_, sum of SUV; SDhist, standard deviation from a histogram; Energy_Hist_, energy from a histogram; Entropy_Hist_, entropy from a histogram; GLCM, gray-level cooccurence matrix; SRE, short-run emphasis; LRE, long-run emphasis; LGRE, low gray-level run emphasis; HGRE, high gray-level run emphasis; SRLGE, short-run low gray-level emphasis; SRHGE, short-run high gray-level emphasis; LRLGE, long-run low gray-level emphasis; LRHGE, long-run high gray-level emphasis; GLNUr, gray-level non-uniformity for run; RLNU, run-length non-uniformity; RP, run percentage; SZE, short-zone emphasis; LZE, long-zone emphasis; LGZE, low gray-level zone emphasis; HGZE, high gray-level zone emphasis; SZLGE, short-zone low gray-level emphasis; SZHGE, short-zone high gray-level emphasis; LZLGE, long-zone low gray-level emphasis; LZHGE, long-zone high gray-level emphasis; GLNUz, gray-level non-uniformity for zone; ZSNU, zone-size nonuniformity; ZP, zone percentage; NGLDM, neighborhood gray-level different matrix; Coarseness_NGLDM_, coarseness from a NGLDM; Contrast_NGLDM_, contrast from a NGLDM; Busyness_NGLDM_, busyness from a NGLDM*.

**Figure 3:**
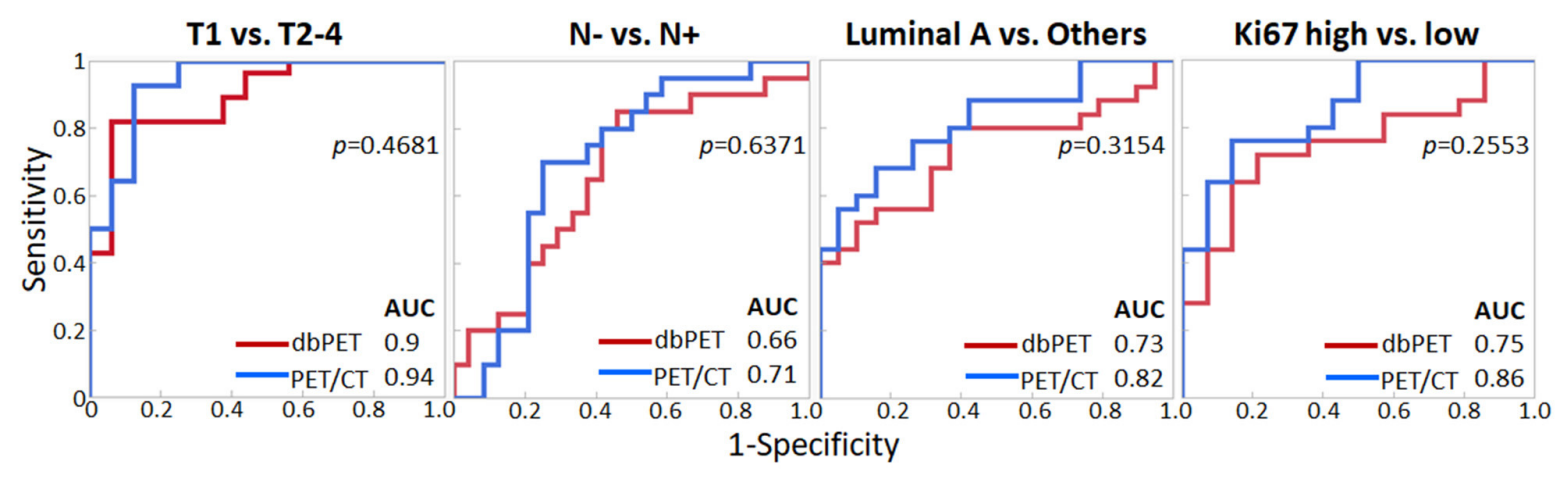
Comparison of ROC analysis for classifying breast cancer characteristics by texture features of dbPET (red line) and whole-body PET/CT (blue line). The AUC values in PCA with texture features of both dbPET and PET/CT were not significantly different. AUC, area under the curve; dbPET, dedicated breast positron emission tomography; CT, computed tomography; PCA, principal component analysis; ROC, receiver-operating-characteristic.

We decided to use the first PCs with eigenvalues >1. As a result, 5 PCs were used for each of dbPET and whole-body PET/CT, and they explained 94 and 92.2% of the variance in dbPET and whole-body PET/CT, respectively. Scree plots and factor loadings of the PCs in the PCA of texture features are shown in Supplementary Figure 1 and Supplementary Table 1, respectively. The PCs of the 38 textural features obtained from dbPET and PET/CT using PCA were excellent in predicting T-category (AUC = 0.89 and 0.94, respectively) and good to fair in predicting N-category (AUC = 0.66 and 0.71), molecular subtype (AUC = 0.73 and 0.82), and Ki67 levels (AUC = 0.75 and 0.86, Table 3). The ROC curves of dbPET and PET/CT, shown in Figure 3, were similar, and there was no statistically significant difference in predictive power between them.

**Table 3:** ROC analysis for classification of tumor characteristics by texture features using principal component analysis.

	**Tumor characteristic**		**AUC**	**Sensitivity**	**Specificity**	**Accuracy**
dbPET	T-category	T1 vs. T2-4	0.89	0.82	0.94	0.86
	N-category	Negative vs. Positive	0.66	0.85	0.54	0.68
	Molecular subtype	Luminal A vs. Others	0.73	0.52	0.89	0.68
	Ki67 level	20%> vs. 20% ≤	0.75	0.72	0.79	0.74
PET/CT	T-category	T1 vs. T2-4	0.94	0.93	0.88	0.91
	N-category	Negative vs. Positive	0.71	0.70	0.75	0.73
	Molecular subtype	Luminal A vs. Others	0.82	0.68	0.84	0.75
	Ki67 level	20%> vs. 20% ≤	0.86	0.76	0.86	0.79

*ROC, receiver operating characteristic; dbPET, dedicated breast positron emission tomography; PET/CT, positron emission tomography/computed tomography; AUC, area under the curve*.

Table 4 summarizes the associations between conventional PET parameters and tumor characteristics. MTV and TLG of both dbPET and PET/CT were associated with the T-category. TLG of PET/CT was associated with tumor subtype; however, no other parameters were associated with BC characteristics (Table 4).

**Table 4:** Associations (*p-value*) between conventional PET parameters and tumor characteristics.

	**dbPET**				**PET/CT**			
**Tumor characteristic**	**T-category**	**N-category**	**Molecular subtype**	**Ki67 level**	**T-category**	**N-category**	**Molecular subtype**	**Ki67 level**
	**Tis-1 vs. 2-4**	**Negative vs. Positive**	**Luminal A vs. Others**	**>20% vs**. **≤20%**	**Tis-1 vs. T2-4**	**Negative vs. Positive**	**Luminal A vs. Others**	**>20% vs. 20%≤**
SUV_max_	0.053	0.0886	0.078	0.0901	0.1078	0.3402	0.0666	0.0741
MTV	<0.0001^*^	0.9295	0.078	0.4309	0.0208^*^	0.7691	0.2873	0.3797
TLG	<0.0001^*^	0.456	0.0504	0.2253	0.0077^*^	0.4484	0.0376^*^	0.0841

* *Statistically significant. dbPET, dedicated breast positron emission tomography; PET/CT, positron emission tomography/computed tomography; SUVmax, maximum standardized uptake value; MTV, metabolic tumor volume; TLG, total lesion glycolysis*.

## Discussion

This is the first study comparing the texture features derived from PET images of BC between dbPET and whole-body PET/CT to the best of our knowledge. Although no individual feature among the 38 texture features calculated from dbPET and whole-body PET/CT BC images was significantly associated with all tumor characteristics of interest, the PCs derived by PCA of these texture features obtained with both modalities had good predictive power for T-category, N-category, molecular subtype (Luminal A vs. others), and Ki67 level. Our results suggest that the texture features derived from PET/CT images of histopathologically proven BC, which has enough volume to be successfully extracted, may apply to the evaluation of neoadjuvant chemotherapy and prognosis prediction. However, PET/CT is inferior in spatial resolution to dbPET.

Moscoso et al. have reported that some texture features of dbPET (Dissimilarity, Entropy, Homogeneity, ZP) were associated with tumor size and molecular subtype (22). We calculated and analyzed 38 texture features, including the first- and higher-order statistical features, thus considering spatial position information. Previous experimental and clinical results demonstrated the importance of using higher-order statistical features in texture analysis (23, 24). Our results also demonstrated the good diagnostic ability of texture features for all the tumor characteristics in this study. However, it was impossible to identify specific features as predictors of every tumor characteristic.

Recently, due to the increased interest and number of published studies, several issues on the use of radiomics have emerged. First, because the number of parameters considered has gradually increased, and the analyses have become increasingly complex, it is difficult to determine the most effective features associated with BC characteristics. Second, there is no consensus on the radiomics method using PET images of BC; therefore, the results differ slightly among studies.

In this study, the texture analysis of dbPET could predict BC characteristics with the same accuracy as that of whole-body PET/CT. However, six BCs, all early-stage, were excluded from the whole-body PET/CT analysis as they could not be successfully extracted on PET/CT images. In previous studies comparing dbPET and whole-body PET/CT, the analysis of standard performance differences between the two scanners in phantom tests and some clinical trials have shown the superiority of dbPET over whole-body PET/CT (12, 13). The significance of dbPET may be demonstrated in the diagnosis of early, small BCs. Texture analyses with a large number of early-stage BCs may also show the efficacy of dbPET.

Another issue was that some BCs, even though they could be visually confirmed to be abnormal on dbPET images, were excluded from this study. This issue may suggest a challenge worth future investigation. The issue is to determine the most suitable tumor extraction method (e.g., gradient methods) for dbPET texture analysis instead of the VOI setting's optimal thresholds. The effect of the image reconstruction methods on the texture analysis of dbPET should also be clarified in the future. High-resolution reconstruction of PET/CT images has been reported to change the textural features compared to standard reconstructed clinical PET images (11, 25). It is necessary to assess how the texture features of dbPET change compared to that of the whole-body PET/CT image due to the differences in the reconstruction method.

Our study has several limitations. The primary limitations include the retrospective nature of the study and small cohort. The evaluation of the association between PET parameters and BC characteristics such as molecular subtype, histopathological grade, and Ki-67 expression could, therefore, not be fully conducted in the context of an associated prognosis. We tried to apply some machine learning classifiers (Support Vector Machine and Random Forest). However, a classifier with high generalization performance could not be obtained. Further studies following the accumulation of a larger number of clinical cases are needed. With more data, the utility of texture analysis could be further generalized by creating classifiers with higher generalization performance. Second, the images analyzed in this study were acquired at 60 min for whole-body PET/CT and 90 min for dbPET after FDG injection. FDG uptake in BC lesions is known to increase over time, which might affect the results. However, it is difficult to change the order of the scans in a clinical setting; thus, this issue needs future investigations.

In conclusion, dbPET was overall superior for detection of BC. However, for BCs that could be successfully extracted, whole-body PET/CT showed the equivalent predictive ability of tumor characteristics using texture analysis to that of dbPET.

## Data Availability Statement

The original contributions presented in the study are included in the article/Supplementary Materials, further inquiries can be directed to the corresponding author/s.

## Ethics Statement

The studies involving human participants were reviewed and approved by Kofu Neurosurgical Hospital. The patients/participants provided their written informed consent to participate in this study.

## Author Contributions

Material preparation, data collection, and analysis were performed by YS. Image analysis was performed by KH. Statistical analysis was performed by YS, DT, and SF. The first draft of the manuscript was written by YS and all authors commented on previous versions of the manuscript. All authors read and approved the final manuscript.

## Conflict of Interest

The authors declare that the research was conducted in the absence of any commercial or financial relationships that could be construed as a potential conflict of interest.

## Abbreviations

AUC: area under the curve
AJCC: American Joint Committee on Cancer
BC: breast cancer
CT: computed tomography
dbPET: dedicated breast tomography PET
ER: estrogen receptor
FDG: fluorodeoxyglucose
FOV: field-of-view
GLCM: gray-level co-occurrence matrix
GLRLM: gray-level run length matrix
GLZSM: gray-level zone size matrix
HER2: human epidermal growth factor receptor 2
IHC: immunohistochemistry
ISH: *in situ* hybridization
MTV: metabolic tumor volume
N: lymph node staging
NGLDM: neighborhood gray-level difference matrix
PCA: principal component analysis
PET: positron emission tomography
PR: progesterone receptor
PSF: point-spread-function
ROC: receiver-operating characteristic
SUV: standardized uptake value
TLG: total lesion glycolysis
T: tumor staging
VOI: volume of interest.
