# Supplementary material for: Texture Analysis in the Diagnosis of Primary Breast Cancer: Comparison of High-Resolution Dedicated Breast Positron Emission Tomography (dbPET) and Whole-Body PET/CT
Source: Front Med (Lausanne). 2020 Dec 23;7:603303. doi: 10.3389/fmed.2020.603303 (PMC7793660; doi:10.3389/fmed.2020.603303)
Supplement: Supplementary file 1 [file Data_Sheet_1.docx]

Supplementary Material

# Supplementary Tables

**Table S1. Loadings of the principal components**

1. dbPET

|  | PC1 | PC2 | PC3 | PC4 | PC5 |
| --- | --- | --- | --- | --- | --- |
| Num of Voxels | -0.08555 | 0.58015 | 0.71517 | -0.2874 | -0.16305 |
| SUV_sum_ | -0.15693 | 0.62109 | 0.68013 | -0.27068 | -0.12729 |
| SD_hist_ | 0.75413 | 0.53669 | -0.02275 | 0.36986 | -0.02614 |
| Skewness | 0.41927 | 0.73296 | 0.45973 | 0.13273 | -0.05798 |
| Kurtosis | 0.75233 | 0.53915 | -0.02297 | 0.36948 | -0.02787 |
| Energy_Hist_ | 0.41867 | 0.73332 | 0.45958 | 0.13257 | -0.0584 |
| Entropy_Hist_ | -0.91783 | 0.04116 | -0.04237 | 0.10191 | 0.25372 |
| Homogeneity_GLCM_ | 0.54194 | -0.79905 | 0.00449 | 0.0047 | 0.07479 |
| Energy_GLCM_ | 0.70671 | -0.57488 | 0.07097 | -0.04395 | 0.17108 |
| Correlation_GLCM_ | 0.64269 | -0.70345 | 0.17353 | 0.00449 | 0.12852 |
| Contrast_GLCM_ | -0.74243 | 0.62157 | -0.17482 | -0.01955 | 0.00857 |
| Entropy_GLCM_ | 0.94705 | 0.23029 | -0.00524 | -0.17125 | 0.1132 |
| Dissimilarity_GLCM_ | -0.38624 | -0.68 | 0.3349 | 0.31438 | 0.14537 |
| SRE | 0.75109 | 0.34904 | -0.28856 | -0.28046 | 0.10115 |
| LRE | -0.83334 | -0.31994 | 0.22036 | 0.29775 | 0.15228 |
| LGRE | 0.44272 | 0.75264 | -0.27129 | -0.14129 | -0.11311 |
| HGRE | -0.89792 | -0.29644 | 0.14435 | 0.24592 | 0.07414 |
| SRLGE | -0.91203 | -0.30849 | -0.00017 | 0.14248 | -0.19711 |
| SRHGE | 0.89552 | 0.33809 | 0.02598 | -0.13558 | 0.21377 |
| LRLGE | 0.42362 | -0.84098 | 0.09491 | 0.07381 | 0.11764 |
| LRHGE | -0.77381 | 0.5576 | -0.02863 | 0.03982 | 0.24699 |
| GLNUr | 0.4058 | -0.85118 | 0.0969 | 0.07727 | 0.1087 |
| RLNU | -0.78722 | 0.54102 | -0.02779 | 0.04375 | 0.24331 |
| RP | 0.49432 | -0.79252 | 0.08755 | 0.05996 | 0.15757 |
| SZE | -0.71298 | 0.62407 | -0.02926 | 0.0226 | 0.26136 |
| LZE | 0.8734 | 0.31963 | 0.00289 | 0.32848 | -0.00069 |
| LGZE | 0.74045 | 0.55041 | -0.0336 | 0.37599 | -0.04317 |
| HGZE | -0.90445 | -0.32807 | -0.00761 | 0.14222 | -0.19993 |
| SZLGE | -0.93016 | -0.24681 | 0.14728 | 0.1396 | -0.03135 |
| SZHGE | 0.80106 | 0.26497 | 0.12886 | -0.09594 | 0.35744 |
| LZLGE | 0.50321 | -0.81253 | 0.08963 | 0.01998 | 0.02521 |
| LZHGE | -0.81077 | 0.49506 | -0.02656 | 0.0648 | 0.27121 |
| GLNUz | 0.26272 | -0.85811 | 0.1444 | 0.01891 | -0.0742 |
| ZSNU | -0.90755 | 0.28592 | 0.01681 | 0.1149 | 0.23762 |
| ZP | 0.77157 | -0.26277 | 0.13725 | 0.03432 | 0.38264 |
| Coarseness_NGLDM_ | 0.0908 | 0.85269 | 0.05345 | -0.07973 | 0.34694 |
| Contrast_NGLDM_ | 0.82437 | 0.39244 | -0.05222 | 0.36704 | -0.10416 |
| Busyness_NGLDM_ | 0.58216 | 0.66567 | -0.08606 | 0.38776 | -0.11865 |

1. PET/CT

|  | PC1 | PC2 | PC3 | PC4 | PC5 |
| --- | --- | --- | --- | --- | --- |
| Num of Voxels | 0.78073 | -0.44048 | 0.00169 | 0.3992 | 0.16562 |
| SUV_sum_ | 0.24624 | -0.74601 | 0.13356 | 0.03086 | 0.01644 |
| SD_hist_ | -0.90814 | -0.08826 | 0.2429 | -0.05498 | 0.27307 |
| Skewness | 0.67098 | 0.66542 | -0.15176 | -0.01088 | -0.10828 |
| Kurtosis | 0.78581 | 0.47784 | 0.01126 | 0.03604 | -0.20892 |
| Energy_Hist_ | -0.05852 | 0.86147 | 0.28534 | 0.29666 | -0.15267 |
| Entropy_Hist_ | 0.13478 | -0.88344 | -0.21546 | -0.33336 | 0.12612 |
| Homogeneity_GLCM_ | 0.95666 | -0.00522 | 0.16696 | 0.0162 | -0.03695 |
| Energy_GLCM_ | -0.51096 | 0.5513 | 0.20047 | 0.4612 | -0.19976 |
| Correlation_GLCM_ | 0.65086 | -0.52327 | -0.13095 | -0.23788 | 0.18667 |
| Contrast_GLCM_ | -0.81285 | 0.40862 | 0.23649 | 0.12372 | 0.12751 |
| Entropy_GLCM_ | 0.6714 | -0.69975 | -0.11993 | -0.10557 | 0.12497 |
| Dissimilarity_GLCM_ | -0.89025 | 0.31099 | 0.15768 | 0.05545 | 0.08974 |
| SRE | -0.89772 | 0.03096 | -0.38785 | 0.11358 | 0.03085 |
| LRE | 0.90717 | -0.02943 | 0.3802 | -0.07707 | -0.02444 |
| LGRE | 0.42502 | 0.77858 | 0.14652 | -0.21049 | 0.31521 |
| HGRE | -0.80361 | -0.45913 | 0.33448 | 0.04433 | 0.08792 |
| SRLGE | 0.40606 | 0.7882 | 0.12699 | -0.20132 | 0.33659 |
| SRHGE | -0.8112 | -0.44902 | 0.33065 | 0.04853 | 0.08766 |
| LRLGE | 0.48535 | 0.73571 | 0.21356 | -0.23709 | 0.23922 |
| LRHGE | -0.77056 | -0.49751 | 0.34807 | 0.02856 | 0.08864 |
| GLNUr | 0.86368 | -0.16034 | 0.05176 | 0.43524 | 0.08557 |
| RLNU | 0.76991 | -0.46087 | -0.01127 | 0.39349 | 0.17342 |
| RP | -0.90582 | 0.04561 | -0.37278 | 0.10919 | 0.02389 |
| SZE | -0.81286 | 0.27736 | -0.29565 | 0.13524 | -0.06831 |
| LZE | 0.83349 | 0.08724 | 0.43719 | -0.02043 | -0.1606 |
| LGZE | 0.25193 | 0.77057 | -0.09925 | -0.01799 | 0.54802 |
| HGZE | -0.81498 | -0.39275 | 0.36 | 0.03006 | 0.09933 |
| SZLGE | -0.04097 | 0.65932 | -0.22448 | 0.12712 | 0.56253 |
| SZHGE | -0.83684 | -0.21948 | 0.2788 | 0.0802 | 0.08884 |
| LZLGE | 0.6355 | 0.37225 | 0.49578 | -0.18356 | -0.20037 |
| LZHGE | -0.32708 | -0.74168 | 0.40616 | -0.07497 | 0.1146 |
| GLNUz | 0.81074 | -0.25529 | -0.05896 | 0.47629 | 0.15493 |
| ZSNU | 0.65986 | -0.60551 | -0.11438 | 0.31541 | 0.20227 |
| ZP | -0.89347 | 0.14908 | -0.34305 | 0.12674 | -0.01148 |
| Coarseness_NGLDM_ | -0.68848 | 0.52967 | -0.01925 | 0.07173 | -0.25685 |
| Contrast_NGLDM_ | -0.83377 | 0.14942 | 0.34091 | 0.17511 | 0.2376 |
| Busyness_NGLDM_ | 0.86446 | 0.22814 | 0.15089 | 0.32145 | -0.04665 |

# SUV, standardized uptake value; SUV_sum_, sum of SUV; SDhist, standard deviation from a histogram; Energy_Hist_, energy from a histogram; Entropy_Hist_, entropy from a histogram; GLCM, grey-level cooccurrence matrix; SRE, short-run emphasis; LRE, long-run emphasis; LGRE, low grey-level run emphasis; HGRE, high grey-level run emphasis; SRLGE, short-run low grey-level emphasis; SRHGE, short-run high grey-level emphasis; LRLGE, long-run low grey-level emphasis; LRHGE, long-run high grey-level emphasis; GLNUr, grey-level non-uniformity for run; RLNU, run-length non-uniformity; RP, run percentage; SZE, short-zone emphasis; LZE, long-zone emphasis; LGZE, low grey-level zone emphasis; HGZE, high grey-level zone emphasis; SZLGE, short-zone low grey-level emphasis; SZHGE, short-zone high grey-level emphasis; LZLGE, long-zone low grey-level emphasis; LZHGE, long-zone high grey-level emphasis; GLNUz, grey-level non-uniformity for zone; ZSNU, Zone-size Nonuniformity; ZP, zone percentage; NGLDM, neighborhood grey-level different matrix; Coarseness_NGLDM_, coarseness from a NGLDM; Contrast_NGLDM_, contrast from a NGLDM; Busyness_NGLDM_, busyness from a NGLDM

## Supplementary Figures


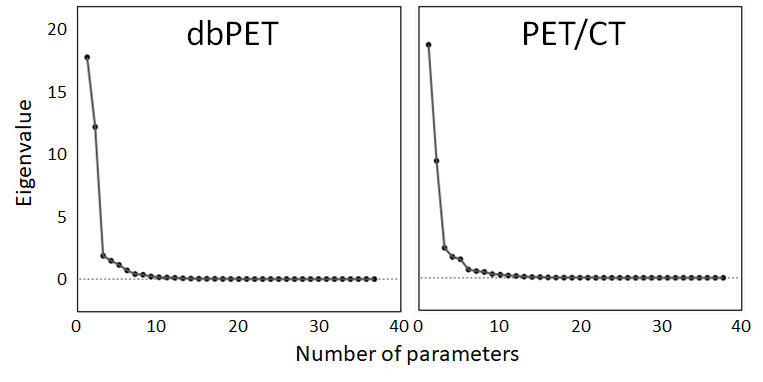


**Supplementary Figure 1.** The eigenvalues' scree plots correspond to each principal component of the texture parameters derived from dbPET and PET/CT BC images.

dbPET, dedicated breast positron emission tomography; PET/CT, positron emission tomography/computed tomography; BC, breast cancer
